# Septins are involved at the early stages of macroautophagy in *S. cerevisiae*

**DOI:** 10.1242/jcs.209098

**Published:** 2018-02-15

**Authors:** Gaurav Barve, Shreyas Sridhar, Amol Aher, Mayurbhai H. Sahani, Sarika Chinchwadkar, Sunaina Singh, Lakshmeesha K. N., Michael A. McMurray, Ravi Manjithaya

**Affiliations:** 1 Molecular Biology and Genetics Unit, Jawaharlal Nehru Centre for Advanced Scientific Research, Jakkur, Bangalore 560064, India; 2 University of Colorado, Anschutz Medical Campus, Department of Cell and Developmental Biology, Aurora, CO 80045, USA

**Keywords:** Autophagy, Noncanonical ring, Septin, Autophagosome biogenesis, Pre-autophagosomal structure, PAS, Atg9 trafficking

## Abstract

Autophagy is a conserved cellular degradation pathway wherein double-membrane vesicles called autophagosomes capture long-lived proteins, and damaged or superfluous organelles, and deliver them to the lysosome for degradation. Septins are conserved GTP-binding proteins involved in many cellular processes, including phagocytosis and the autophagy of intracellular bacteria, but no role in general autophagy was known. In budding yeast, septins polymerize into ring-shaped arrays of filaments required for cytokinesis. In an unbiased genetic screen and in subsequent targeted analysis, we found autophagy defects in septin mutants. Upon autophagy induction, pre-assembled septin complexes relocalized to the pre-autophagosomal structure (PAS) where they formed non-canonical septin rings at PAS. Septins also colocalized with autophagosomes, where they physically interacted with the autophagy proteins Atg8 and Atg9. When autophagosome degradation was blocked in septin-mutant cells, fewer autophagic structures accumulated, and an autophagy mutant defective in early stages of autophagosome biogenesis (*atg1Δ*), displayed decreased septin localization to the PAS. Our findings support a role for septins in the early stages of budding yeast autophagy, during autophagosome formation.

This article has an associated First Person interview with the first author of the paper.

## Abstract (precis)

**Highlighted Article:** Septin proteins, which form cytoskeletal filaments and bind membranes, are required for efficient assembly of functional autophagic structures in budding yeast cells.

## INTRODUCTION

Macroautophagy (herein autophagy) is an evolutionarily conserved intracellular waste disposal and recycling process that is critical for normal cellular and organismal homeostasis. Autophagy involves the formation of double-membrane vesicles called autophagosomes that engulf intracellular material destined for degradation. Autophagosomes eventually fuse with vacuoles or lysosomes, resulting in cargo degradation and recycling of cellular building blocks, such as amino acids, back to the cytoplasm. The biogenesis of autophagosomes remains incompletely understood.

In budding yeast cells, the site of autophagosome formation is known as the pre-autophagosomal structure (PAS) and is perivacuolarly located. Recent work has shown that the PAS is tethered to endoplasmic reticulum (ER) exit sites where multiple autophagy proteins colocalize in a hierarchical sequence (Graef et al., 2013; Suzuki et al., 2007). The membrane source for the developing autophagosome is contributed by the trafficking of Atg9 along with its transport complex (Atg1–Atg11–Atg13–Atg23–Atg27–Atg2–Atg18–TRAPIII) to help build the initial cup-shaped structure, the phagophore (Legakis et al., 2007; Reggiori et al., 2004; Tucker et al., 2003). Additional recruitment of the Atg5–Atg12–Atg16 complex as well as Atg8 allows the completion of the autophagosome (Feng et al., 2014).

Septin proteins bind guanine nucleotides and co-assemble in hetero-oligomers capable of polymerizing into cytoskeletal filaments (Mostowy and Cossart, 2012). Septin filaments associate directly with membranes in a curvature-dependent manner (Bridges et al., 2016) and regulate membrane dynamics, including vesicle fusion events (Mostowy and Cossart, 2012). In immune cells, septins also localize transiently to the phagocytic cup and are functionally involved in phagocytosis (Huang et al., 2008). Septins have been implicated in autophagy in mammalian cells infected by intracellular bacteria, where they form cage-like structures around the bacterial cells that colocalize with the autophagosome marker autophagosome marker MAP1LC3A, the homolog of yeast Atg8. It is believed that these structures entrap bacteria, restricting their motility and targeting them for autophagy-mediated degradation (Mostowy et al., 2009, 2010). During *Shigella* infection, assembly of septin cages and the autophagosome in the host mammalian cells are interdependent (Mostowy et al., 2010, 2011; Sirianni et al., 2016). Despite these findings, it remains unclear to what extent septins contribute to autophagy outside the context of bacterial infection (Torraca and Mostowy, 2016).

In *S. cerevisiae* cells undergoing mitotic proliferation, five septin proteins – Cdc3, Cdc10, Cdc11, Cdc12 and Shs1 – comprise an array of filaments that is directly associated with the plasma membrane at the mother–bud neck, and controls cell polarity, bud morphogenesis and cytokinesis (Glomb and Gronemeyer, 2016; Oh and Bi, 2011). Upon nitrogen starvation, diploid yeast cells undergo meiosis and sporulation, during which a cup-shaped double-membrane structure, the prospore membrane (PSM), engulfs haploid nuclei and other organelles to form stress-resistant spores (Neiman, 2005, 2011). Yeast septins are required for proper PSM biogenesis (Heasley and McMurray, 2016), but there was no known role for septins in yeast autophagy. Here, we describe autophagy defects in septin-mutant strains and physical interactions between septins and established autophagy factors that support a functional role for septins in yeast autophagy.

## RESULTS

### Autophagy defects in septin mutants

**Fig. 1.**
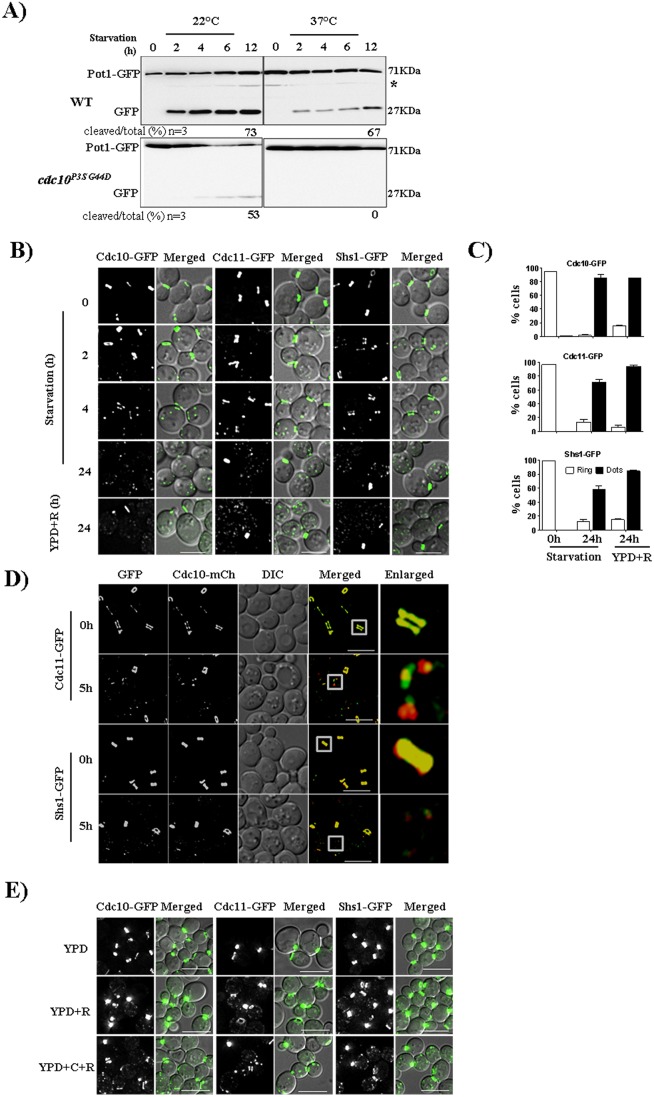
**Septins migrate from the pre-existing bud-neck ring to cytoplasm during starvation.** (A) Pexophagy was affected in *cdc10^P3SG44D^* (*cdc10-5*) cells as compared to WT cells at the non-permissive temperature (37°C). (B) Microscopy images of Cdc10–GFP, Cdc11–GFP and Shs1–GFP cells under nutrient rich, nutrient deficient and rapamycin (0.4 µg/ml) treatment conditions. Cells from log phase (0.6 to 0.8 OD) were transferred to starvation medium (1 OD/ml) and imaged at different time points. (C) Quantification of the number of cells showing rings and puncta grown in rich, starvation or rapamycin treatment medium for 24 h. For quantification, cells showing only ring or only dots were considered. Images acquired were converted into maximum intensity projections, deconvolved and a total of 100 cells were quantified. (D) Cdc10, Cdc11 and Shs1 all colocalize as puncta during starvation. Strains JTY5396 and JTY5397 were grown as in B and imaged. (E) Septin localization in presence of cycloheximide (C) and rapamycin (R). Cells were grown as described in Fig. 1B in presence of cycloheximide (50 µg/ml) and rapamycin (YPD+C+R) and in presence of rapamycin (0.4 µg/ml) alone (YPD+R). Scale bars: 5 µm.

To identify autophagy defects in viable mutant yeast strains, we introduced into a collection of temperature-sensitive (Ts^−^) mutants in a *POT1-GFP* strain, which expresses a marker of pexophagy (Kondo-Okamoto et al., 2012), a specialized form of autophagy in which peroxisomes are degraded (Oku and Sakai, 2016). Targeting of Pot1–GFP to the vacuole during starvation-induced pexophagy results in destruction of the Pot1 part of the fusion protein and accumulation of free GFP, which is readily detected by immunoblotting (Fig. 1A; Fig. S1A,B). Unlike in wild-type (WT) cells, where free GFP accumulated at both 22°C and 37°C, in cells expressing any of several Ts^−^ mutant alleles of the septin *CDC10* (G100E or P3S G44D) or *CDC11* (G29E, G34D or S31F S100P) more free GFP was detected at 22°C, compared to what was seen at 37°C, and the Pot1–GFP fusion remained intact at 37°C (Fig. 1A; Fig. S1A). These results were also corroborated by using fluorescence microscopy to visualize the delivery of GFP-labeled peroxisomes to the vacuole as diffuse GFP inside the vacuolar lumen (Fig. S1B). At 37°C the number of starved septin-mutant cells showing free GFP inside the vacuole was reduced significantly when compared to the numbers of starved WT cells, and also when compared to numbers of mutant cells incubated at 22°C (Fig. S1C). These data point to a requirement for septin function in pexophagy.

In nutrient-replete conditions, the Ts^−^ mutants of *CDC10* and *CDC11* in which we found pexophagy defects arrest cell division with failed cytokinesis (Hartwell, 1971). Interestingly, we did not observe Pot1–GFP-processing defects in cells expressing Ts^−^ mutant versions of *CDC3* (G365R) or *CDC12* (G247E) (Fig. S1D), which were originally isolated in the same cell division screen (Hartwell, 1971) as the *CDC10* and *CDC11* mutants that caused pexophagy defects. To explain this discrepancy, we considered that in *cdc3(G365R)* or *cdc12(G247E)* cells, high temperature prevents *de novo* assembly of septin complexes but does not destabilize existing structures (Dobbelaere et al., 2003; Kim et al., 1991; Weems et al., 2014). Since pexophagy, like autophagy in general, occurs in starved non-dividing cells, we hypothesized that a functional contribution of septins to pexophagy may not require assembly of new septin complexes, and instead utilizes pre-existing complexes assembled prior to the nutrient withdrawal and temperature upshift. Indeed, yeast septins are exceedingly long-lived proteins, even during starvation (McMurray and Thorner, 2008). In support of this model, Pot1–GFP processing was compromised in *cdc12-td* cells (Fig. S1D), which express a temperature-degron-tagged Cdc12 that is known to cause rapid disassembly of pre-existing filamentous septin structures associated with the bud neck in dividing cells (Weems et al., 2014). These findings indicate that the septin complexes involved in pexophagy are composed of the same septins that were previously synthesized when nutrients were available and supported cytokinesis in budding cells.

To ask whether septins are more generally involved in autophagy, we examined the processing of GFP–Atg8, which is processed in the vacuole during autophagy (Cheong and Klionsky, 2008). Similar to the pexophagy results obtained with Pot1–GFP, we noticed considerable slowdown of autophagic flux in septin mutant cells, as evidenced by slower processing of GFP–Atg8 (Fig. S2). In addition to mutant alleles harboring substitutions in specific residues, conditionally viable septin-mutant cells can be obtained by deleting the *CDC10* gene (Flescher et al., 1993; Frazier et al., 1998; McMurray et al., 2011). In cells lacking Cdc10, septin filament assembly and functions essential for mitotic proliferation require septin hetero-hexamers formed via non-native Cdc3 homodimerization (McMurray et al., 2011). Cells lacking Cdc10 are temperature-sensitive for mitotic proliferation due to inefficient Cdc3 homodimerization at high temperatures (McMurray et al., 2011). If the same septin complexes that function in cytokinesis are also involved in autophagy, then we would expect to find autophagy defects in *cdc10*Δ cells at 37°C. Indeed, this was the case, as assayed by both Pot1–GFP and GFP–Atg8 processing (Fig. S3A,B). Similarly, cells lacking Cdc11 require non-native Cdc12 homodimerization for survival, but septin function is more severely compromised in *cdc11*Δ mutants because Shs1 occupies the same position as Cdc11 in a subset of hetero-octamers and, in doing so, prevents efficient Cdc12 homodimerization (McMurray et al., 2011). We found that Pot1–GFP processing was more severely compromised in *cdc11*Δ than in *cdc10*Δ mutants (Fig. S3A,B), consistent with the relative magnitude of the effects on mitotic proliferation. Also consistent was the relatively minor effect of deleting *SHS1* (Fig. S3A,B), which has only mild effects on mitotic proliferation at 37°C (Finnigan et al., 2015; Mino et al., 1998). Finally, deleting *SHS1* in a *cdc11*Δ background improves mitotic proliferation, presumably because Shs1 no longer interferes with Cdc12 homodimerization (McMurray et al., 2011). Pexophagy and autophagy defects in *cdc11*Δ *shs1*Δ cells were equivalent to the defects in *cdc10*Δ cells (Fig. S3A,B), providing additional support for our conclusion that cytokinesis and autophagy share similar requirements for septin complex assembly.

In WT cells, the induction of autophagy triggers a coalescence of multiple small vacuoles into a single organelle (Baba et al., 1994), which is readily observed by visualizing FM4-64 labeling of the vacuolar membrane (Fig. S3C). We noticed that in septin mutant cells, particularly the viable deletion mutants, vacuolar coalescence was largely defective (Fig. S3C). The defect in pexophagy in *cdc11*Δ cells was rescued by introduction of a plasmid encoding WT Cdc11, confirming that the pexophagy defect resulted from the absence of Cdc11 (Fig. S3E).

If septin mutant cells have defects in autophagy, then they should be sensitive to starvation, survival during which requires a functional autophagy pathway (Suzuki et al., 2011). Indeed, heterozygous diploid strains lacking one copy of *CDC10* are known to be sensitive to nutrient deprivation (Davey et al., 2012), a previously unexplained phenotype. Haploids lacking Cdc10 are also sensitive to rapamycin (Chan et al., 2000), which could reflect a requirement for autophagy to survive the starvation-like metabolic conditions that result from inhibition of Tor1/2, as autophagy is induced by rapamycin even in nutrient-replete conditions (Noda and Ohsumi, 1998). Finally, we note that a high-throughput genetic interaction study previously reported negative interactions between *cdc10* mutants and mutations in the autophagy genes *ATG3*, *ATG8* and *ATG9* (Costanzo et al., 2010). These findings provide independent support for a functional requirement for septins in autophagy.

### Septins move from the bud neck to the PAS during starvation and associate with mature autophagosomes

Our genetic findings indicate a functional role for septin complexes in autophagy and further suggested that the same septin complexes assembled prior to induction of autophagy are utilized during autophagy, without a requirement for new septin synthesis or assembly. Based on these findings, we predicted that, during starvation, septins should re-localize from the bud neck to sites of autophagosome assembly (i.e. the PAS). Consistent with this prediction, upon starvation GFP-tagged Cdc10, Cdc11 and Shs1 present at the bud neck quickly (within 5 h) transitioned to cytosolic puncta (Fig. 1B,C; Movie 1). Addition of rapamycin to cells in rich medium had the same effect (Fig. 1B,C). mCherry-tagged Cdc10 colocalized in these cytosolic puncta with Cdc11–GFP and Shs1–GFP (Fig. 1D). We could not obtain conclusive results with fluorescently tagged Cdc3 or Cdc12 due to the propensity in starvation conditions of these fusion proteins to form, and incorporate other septins into, aberrant rod-shaped structures (Fig. S1E; data not shown). Crucially, relocalization of septins from the bud neck to cytosolic puncta did not require new protein synthesis, since equivalent results were observed in the presence of the translation inhibitor cycloheximide (Fig. 1D). These findings demonstrate that, upon starvation, pre-existing septin proteins re-localize from the site of cytokinesis to cytosolic puncta, consistent with a role in a cytosolic process in these conditions.

**Fig. 2.**
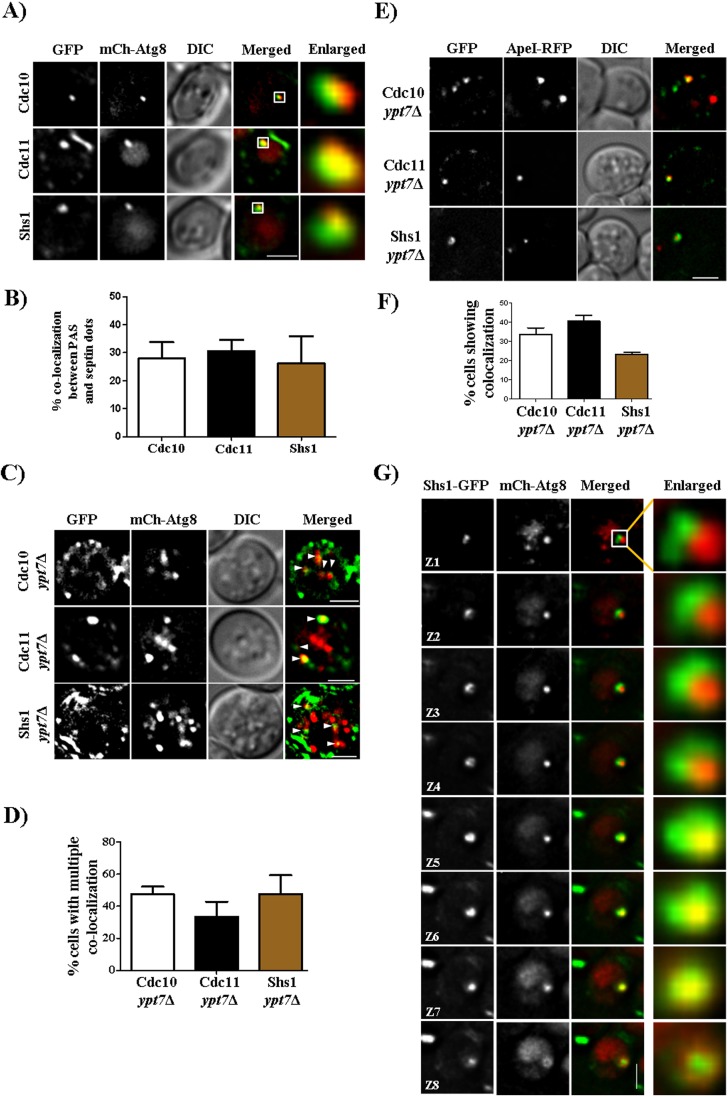
**Septins colocalize with autophagosomes and form ring like structures.** (A) Septins colocalize with the mCherry–Atg8 (mCh-Atg8)-labeled PAS. Cells were grown as described in Fig. 1B and imaged. All images are of a single *z*-section and are deconvolved. (B) Quantification of the number of PASs that colocalize with septins. More than 300 cells were counted. From these cells, only 20–30% cells showed a PAS and cells that showed colocalization between the PAS and septin dot were quantified. Quantification was performed manually by using the cell counter plugin of Fiji at every *z*-section. (C) Representative images showing colocalization of septins and autophagosomes (autophagosomes are highlighted by white arrowheads). Cells were grown as in Fig. 1B and were imaged. (D) Quantification of the number of cells showing multiple colocalizations between septins and mCherry–Atg8 puncta. For quantification, images were deconvolved and background subtracted, and then colocalization was checked by using the colocalization highlighter plugin. Colocalized points were then quantified by using the cell counter plugin of Fiji. More than 150 cells were quantified. (E) Colocalization of septins with Ape1–RFP. Cdc10-GFP, Cdc11–GFP and Shs1–GFP *ypt7*Δ cells expressing Ape1–RFP were grown as in Fig. 1B and imaged. (F) Quantification of the number of septin puncta colocalized with Ape1–RFP puncta. (G) Formation of a non-canonical ring around mCherry–Atg8 by Shs1–GFP. Cells were grown as in Fig. 1B and were imaged. Eight z-sections of the same image at 0.2 µm each are shown. Scale bars: 2 µm.

To ask whether the cytosolic puncta to which septins relocalize upon starvation included the PAS, we examined septin–GFP localization in cells also expressing an mCherry-tagged version of Atg8, which marks the PAS prior to its processing within the vacuole (Delorme-Axford et al., 2015). Approximately 30% of cytosolic septin–GFP puncta in starved cells were also labeled by mCherry–Atg8 (Fig. 2A,B). Whereas in WT cells autophagosomes disappear as they fuse with the vacuole, in cells lacking Ypt7, a Rab GTPase required for autophagosome-vacuole fusion, autophagosomes persist (Ishihara et al., 2001; Kim et al., 1999). We observed a corresponding increase in the number of septin foci that were also marked by mCherry–Atg8 in cells lacking Ypt7 (Fig. 2C,D). Similar results were obtained using GFP-tagged septins and an RFP-tagged version of Ape1/Lap4, an aminopeptidase that is tethered to the PAS in its precursor form prior to proteolytic activation in the vacuolar lumen (Delorme-Axford et al., 2015) (Fig. 2E,F). Careful imaging revealed that septin–GFP puncta could often be resolved as rings surrounding the PAS (Fig. 2G; Fig. S4A–E). The dimensions of these rings (400–600 nm in diameter) are about half the size of septin rings at the bud neck (Okada et al., 2013) and are instead similar to the size of autophagosomes (400–900 nm in diameter) (Suzuki and Ohsumi, 2010). The autophagy protein Atg9 is also known to form ∼500-nm-wide rings around mCherry–Atg-marked autophagosomes (Yamamoto et al., 2012). Septin–GFP localization at the PAS was transient (Movie 2), providing an explanation for our observations that not every PAS was associated with septins. These results support a model in which septins associate with the developing PAS and remain associated with mature autophagosomes.

### Septins interact with autophagy proteins

**Fig. 3.**
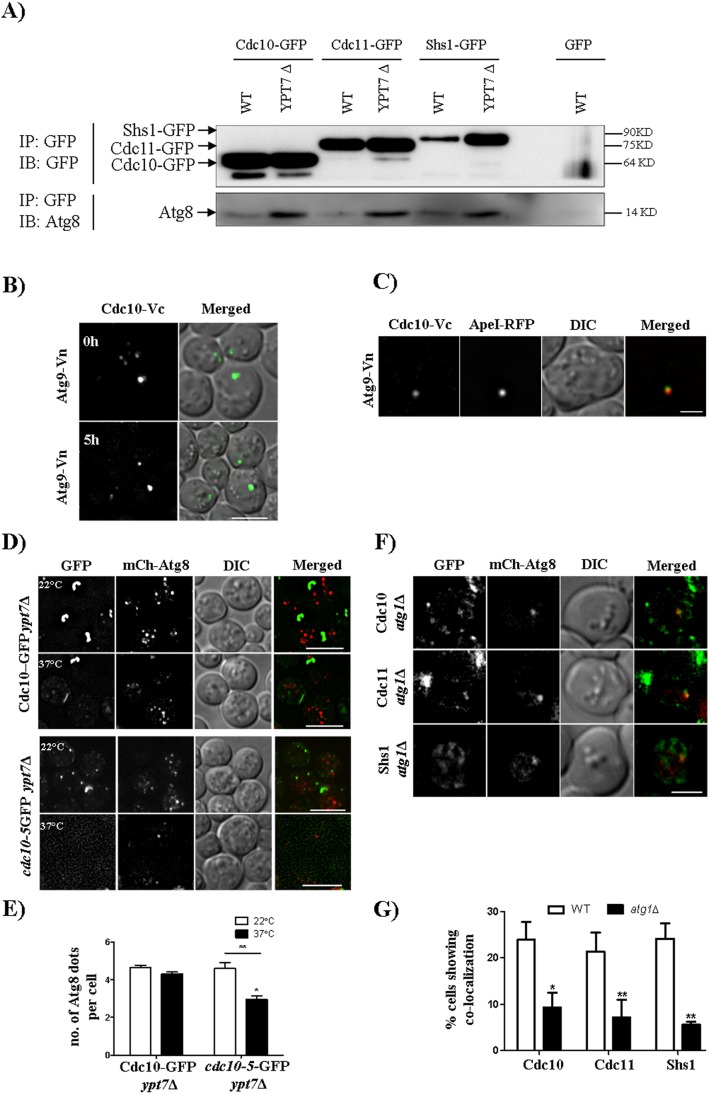
**Septins are involved in autophagosomes biogenesis.** (A) Western blot showing the septin–Atg8 interaction in WT and in *ypt7*Δ strains expressing Cdc10–GFP, Cdc11–GFP and Shs1–GFP. Cells were grown as described in the Materials and Methods. IB, immunoblot; IP, immunoprecipitation. (B,C) BiFC experiments. A strain expressing Cdc10-Vc (Cdc10 C-terminus tagged with C-terminus of Venus) and Atg9-Vn (Atg9 C-terminus tagged with N-terminus of Venus) with or without Ape1–RFP was grown as described in Fig. 1B and imaged after 5 h of incubation in starvation medium. (D) Representative images and (E) quantification showing autophagosome number per cell at 37°C. All the images are maximum intensity projections, and more than 50 cells were quantified manually with Fiji. *P<0.05 (comparison between non-Ts and Ts at 37°C); **P<0.01 (comparison between 22°C and 37°C in Ts) (two-way ANOVA). (F) Representative images and (G) quantification of colocalization events between mCherry–Atg8 and the three GFP-tagged septins in the *atg1*Δ strain. A total of 50 cells were quantified manually at every *z*-plane. *P<0.05 for Cdc10–GFP, **P<0.01 for Cdc11–GFP and Shs1–GFP (two-way ANOVA). Scale bars: 2 µm (C,F); 5 µm (B,D).

To ask whether septin localization at the PAS involves physical interactions between septins and known autophagy proteins, we employed two parallel approaches. Immunoprecipitation of GFP-tagged septins using the GFP tag as an epitope resulted in the co-precipitation of untagged Atg8, and the amount of co-precipitated Atg8 increased in the absence of Ypt7 (Fig. 3A), consistent with a prolonged interaction due to stabilization of autophagosomes. Negligible Atg8 was precipitated by the GFP antibody when GFP was not fused to a septin (Fig. 3A). Furthermore, Cdc10 interacted *in vivo* with Atg9 in a bimolecular fluorescence complementation (BiFC) assay (Fig. 3B); other septins were not tested. In addition to single cytoplasmic puncta in starved cells, which we confirmed to be the PAS because they colocalized with Ape1–RFP, a Cdc10–Atg9 BiFC signal was observed at the necks of budding cells (Fig. 3B,C; Movie 3). Since Atg8 and Atg9 are also involved in the ‘cytoplasm to vacuole targeting’ (Cvt) pathway, a biosynthetic form of selective autophagy active even in rich medium conditions (Reggiori and Klionsky, 2013), these observations likely represent otherwise transient associations between septins and the autophagy machinery during Cvt that are prolonged by the essentially irreversible BiFC event. The autophagy proteins are thereby artificially tethered to the septin ring at the bud neck, which facilitates detection of the BiFC event, but does not faithfully report on where the interaction first took place. Taken together, these findings provide strong evidence that septins physically interact with the core autophagy machinery both during Cvt and starvation-associated autophagy.

### Septins are involved in autophagosome biogenesis

To determine at which stage of autophagosome assembly septins function, we first examined septin-mutant cells to identify the step in autophagosome formation that fails when septins are dysfunctional. We combined the *cdc10(P3S G44D)* mutation with *ypt7*Δ, to block autophagosome degradation, and shifted starved cells to 37°C. We found a decrease in the number of mCherry–Atg8 foci per cell compared to that in *ypt7*Δ cells with WT septins (Fig. 3D,E), indicating that septin dysfunction perturbs autophagosome biogenesis, rather than delivery of autophagosomes to the vacuole and degradation. Next, we examined septin–GFP and mCherry–Atg8 localization in cells lacking Atg1, in which PAS assembly begins but no mature autophagosomes are produced (Suzuki et al., 2007). In *atg1*Δ cells, colocalization between GFP-tagged septins and mCherry–Atg8 decreased significantly compared to that in WT cells (Fig. 3F,G), suggesting that septins arrive at the nascent autophagosome after the PAS has already begun to assemble.

**Fig. 4.**
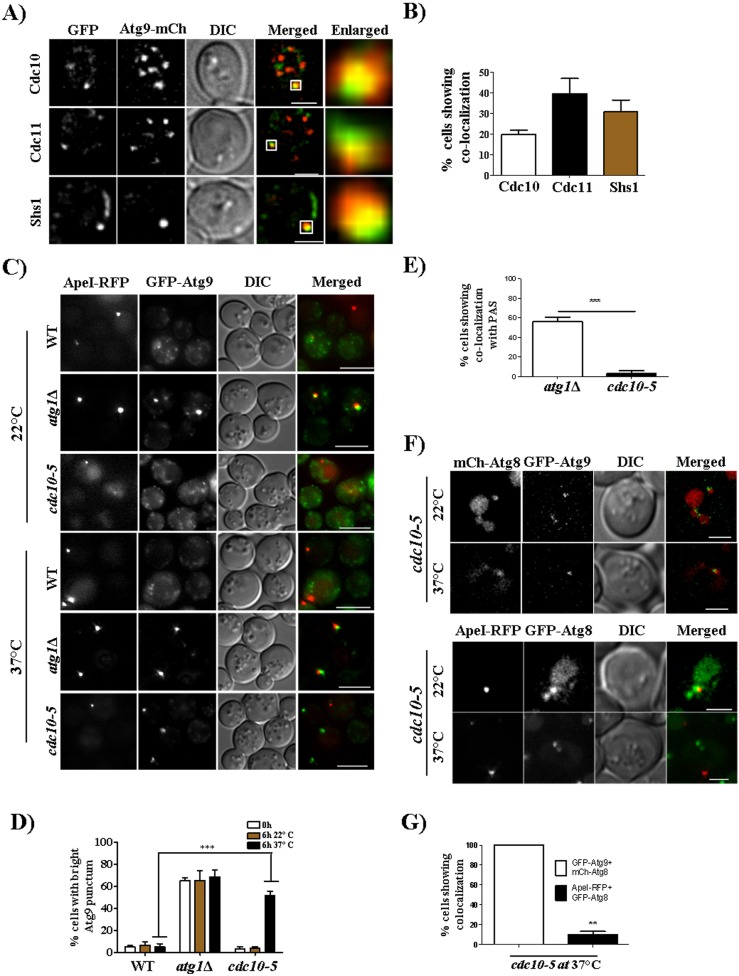
**Septins colocalize with Atg9 and play a role in Atg9 retrograde transport.** (A) Representative images showing colocalization between septins and Atg9. Cdc10–GFP, Cdc11–GFP and Shs1–GFP cells expressing Atg9–mCherry (Atg9-mCh) were grown as in Fig. 1B and were imaged. (B) Quantification of the number of cells showing colocalization between septins and Atg9–mCherry puncta. Quantification was performed as in Fig. 3G. More than 150 cells were quantified. (C) Atg9 retrograde transport is affected in the *cdc10-5* strain at 37°C. Cells were grown in starvation medium for 6 h and were imaged. (D) Quantification of the number of cells showing a bright Atg9 punctum at 22°C (permissive temperature) and 37°C (non-permissive temperature). Quantification was performed manually by using Fiji software, and a total of 50 cells were quantified in each of the three experiments. ***P<0.001, 22°C versus 37°C in WT and *cdc10-5* cells (two-way ANOVA). (E) Quantification of the number of cells showing colocalization between the bright GFP–Atg9 punctum and Ape1–RFP. Quantification was performed manually by using Fiji software at each *z*-section, and a total of 30 cells were quantified in each of the three experiments. ***P<0.001, *atg1*Δ versus *cdc10-5* cells (unpaired t-test.). (F) Colocalization between GFP–Atg9 and mCherry–Atg8, and GFP–Atg8 and Ape1–RFP. The *cdc10-5* cells expressing either GFP–Atg9 with mCherry–Atg8 or GFP–Atg8 with Ape1–RFP were grown in SD Ura or SD −His −Ura medium at 22°C. Logarithmically growing cells were then incubated in starvation medium (1 OD/ml) for 3 h at 22°C and 37°C. (G) Quantitation of the colocalization of GFP–Atg9 and mCherry–Atg8 puncta with GFP–Atg8 and Ape1–RFP puncta. 30 cells were quantified in each of the three experiments. **P<0.01 (paired *t*-test). Scale bars: 2 µm (A,F); 5 µm (C).

The autophagy protein Atg9 accumulates at the PAS in *atg1*Δ cells due to a defect in retrograde Atg9 transport to the sources of membrane trafficking (Sekito et al., 2009). In WT cells, Atg9–mCherry colocalized primarily with GFP-tagged septins (Fig. 4A,B). Interestingly, a single bright punctum of GFP–Atg9 was also observed in *cdc10(P3S G44D)* cells incubated at 37°C (Fig. 4C). This punctum colocalized with the PAS marker Ape1 (Fig. 4C–E), indicating that septin dysfunction prevents proper Atg9 retrograde transport away from the PAS, a phenocopy of the absence of Atg1. We further noticed that Ape1 was mislocalized in septin mutant cells, as only a few Ape1–RFP puncta colocalized with GFP–Atg8 in *cdc10(P3S G44D)* cells incubated at 37°C (Fig. 4F,G). Taken together, these experiments point to a role for septin complexes in biogenesis of autophagosomes following PAS assembly.

## DISCUSSION

The original budding yeast mutants defective in autophagy were identified by unbiased genetic screens based on phenotypes of failure to accumulate ‘autophagic bodies’, to survive during nitrogen starvation (Tsukada and Ohsumi, 1993) or to degrade specific cytoplasmic enzymes (Thumm et al., 1994), with an underlying assumption that autophagy is non-essential for colony growth in rich medium. Another study systematically searched for autophagy defects in a collection of mutants harboring hypomorphic alleles of essential genes (Shirahama-Noda et al., 2013), but by definition these alleles provide sufficient function to support proliferation. We report here the identification of septin mutants in what is, to our knowledge, the first unbiased screen for autophagy (pexophagy) defects among conditionally lethal mutants. We observed severe defects under conditions non-permissive for proliferation, which explains why septin mutants were not isolated in previous autophagy screens.

Septins are functionally important for numerous processes involving changes in membrane shape, ranging from cytokinesis (Hartwell, 1971; Kim et al., 2011) to mitochondrial fission (Pagliuso et al., 2016) to the retraction of membrane blebs (Gilden et al., 2012). Macroautophagy requires the synthesis and directed extension of a double-bilayer ‘isolation membrane’ which, when its leading edges fuse together, becomes an autophagosome, and is destined for fusion with the vacuole or lysosome. In metazoan cells, septins form cage-like assemblies around intracellular bacteria and recruit autophagocytic machinery (Torraca and Mostowy, 2016), but these studies did not determine whether septins participate in the assembly of autophagocytic membrane structures per se, and defects in general autophagy (i.e. not associated with bacterial infection) have not been reported.

The Atg9 retrograde transport defects we found in septin mutants suggest that septins may help Atg9 molecules to deliver membrane source for developing autophagosomes. Additionally, our findings of dynamic septin localization to a subset of PAS structures after initial PAS formation, septin rings of diameters consistent with those of the autophagosomal membranes, interactions between septins and the autophagosomal membrane protein Atg9, and defects in autophagosome maturation in septin mutants are all consistent with roles for septins in guiding isolation membrane extension. In this regard, the autophagy defects we observed in septin mutant cells are reminiscent of defects in extension of the yeast PSM (Heasley and McMurray, 2016), another double-bilayer membrane that engulfs cytoplasmic components prior to fusion of its leading edges. Notably, whereas proper septin function in PSM extension appears to require the *de novo* assembly of hetero-octamers containing two sporulation-specific septin proteins (Garcia et al., 2016), our findings suggest that the same septin hetero-octamers assembled during mitotic proliferation are sufficient to support autophagosome maturation. Emerging studies (Bridges et al., 2016) suggest that the ability of rod-shaped septin hetero-oligomers to interact with membranes of specific micron-scale curvatures and polymerize into filaments may be key to septin function in various contexts. The details of interactions between septins and the established autophagy machinery, particularly membranes, and whether post-translational modifications to the septins drive their departure from the site of cytokinesis are compelling subjects for future research.

## MATERIALS AND METHODS

### Yeast strains and media

Wild-type (WT) and autophagy knockout mutant yeast strains used in this study are derived from BY4741, BY4742, and S288C. These strains were obtained from EUROSCARF. Strain and primer details are listed in Tables S1 and S2, respectively. The WT Pot1–GFP strains are laboratory strains with GFP tagged genomically to the C-terminus of Pot1, and were obtained from Prof. Richard Rachubinski, University of Alberta, Canada. Septin Ts^−^ mutants were kindly provided by Prof. Charlie Boone, Toronto, into which a *POT1-GFP* cassette was transformed to obtain strains used for pexophagy assays. Septin knockout mutants were prepared using the standard transformation protocols (Baudin et al., 1993). GFP-ATG8 pRS316 and 2xmCherry-ATG8 pRS316 plasmids were a kind gift from Prof. Yoshinori Ohsumi, Tokyo Institute of Technology, Tokyo. GFP-Atg9 pRN295 was a kind gift from Prof. Michael Thumm, University of Stuttgart, Germany. Vector pSUN5 was created by amplifying Atg9 promoter and the open-reading frame (ORF) from a WT strain and cloned into the pRS316vector at the SacII and NotI sites. The tandem repeat of mCherry separated by a 45 bp linker region was cloned in two steps between the Not1, XmaI and HindIII sites. A linker region of 13 bp was also included between the Atg9 ORF and start codon of mCherry.

WT cells and mutants were grown in YPD medium (1% yeast extract, 2% peptone and 2% dextrose) at 30°C and 22°C, respectively. For pexophagy assays, oleate medium (0.25% yeast extract, 0.5% peptone, 1% oleate, 5% Tween-40 and 5 mM phosphate buffer) was used to induce peroxisome formation, and synthetic defined medium (0.17% yeast nitrogen base lacking amino acids and ammonium sulphate plus 2% dextrose) was used to induce autophagy. For Ts^−^ mutants and knockout mutants, 22°C and 37°C were used as the permissive and non-permissive temperatures, respectively.

### Microscopy and cytology

Cells were grown in respective media, centrifuged (20817 ***g*** for 2 min). and were mounted on agarose (2% w/v) pads for microscopy. Images were taken in *z*-sections of 0.2 µm step size using a Delta Vision microscope (GE Healthcare) fitted with 100×1.4 NA objective and Cool-SNAP HQ2 camera. Images were acquired using FITC and TRITC filters. Image processing and quantification were performed with SoftWorx (GE Healthcare) and Fiji (NIH) software. For colocalization analysis, images were de-convolved and background subtracted, and colocalized entities were either quantified manually by using the cell counter plugin or automatically by using the colocalization highlighter plugin in Fiji for all *z*-sections. Representative colocalizations events quantified manually were also confirmed by line profile and colocalization measurement options in SoftWorx (GE Healthcare). All the supplementary movies are of a single *z*-plane.

### Western blot analysis and quantification

Whole cell extracts were prepared via the trichloroacetic acid (12.5% TCA w/v) precipitation method followed by ice-cold acetone washes (twice). Protein extracts were then analyzed by SDS-PAGE and western blotting (mouse anti-GFP monoclonal 1:3000, cat. no. 11814460001, Roche Applied Science). Blots were visualized using anti-mouse-IgG secondary antibody conjugated to horseradish peroxidase (HRP; Bio-Rad, 1:10,000) on a gel documentation system (G:Box chemi XT4, Syngene). Images were analyzed using ImageJ (NIH). Lanes were marked, followed by plotting and labeling peaks using the analysis tool for gels. The ratios of the intensity of the free GFP band to total GFP (to either the Pot1–GFP plus GFP band, or GFP–Atg8 plus GFP) band was quantified and plotted as the percentage of cleaved product.

### Pexophagy assay

Cells were grown in YPD and 0.2 optical density (OD) units (measured at 600 nm) was inoculated in fresh YPD medium. To induce peroxisome formation, cells were then incubated in oleate medium (1 OD/ml) for 14 to 16 h. Cells were then washed twice with sterile water and SD-N medium was added (3 OD/ml) to induced pexophagy. Time points were collected for western blot analysis. For microscopy, the 6-h time point was collected and FM4-64 (1 µl/ml of 1 mg/ml stock) was added to stain the vacuoles at respective temperatures. Cells were then imaged.

### Immunoprecipitation

WT and *ypt7*Δ expressing Cdc10–GFP, Cdc11–GFP or Shs1–GFP together with mCherry–Atg8 were grown in SD-Ura medium. After the cultures reached 0.6–0.8 OD, 400 OD cells were transferred to starvation medium (3 OD/ml) and were incubated for 5 h at 30°C. After 5 h, 100 OD cells were lysed as per the protocol mentioned in Nagaraj et al. (2008). For immunoprecipitations, 15 µl of GFP-trap beads (Chromotek) were added and the manufacturer's protocol was followed. After the immunoprecipitation, the sample containing the beads was heated and loaded on the gel for SDS-PAGE followed by western blotting. Blots were first probed with either mouse anti-GFP (1:3000, cat. no. 11814460001, Roche Applied Science) or rabbit anti-Atg8 antibody (1:3000, a kind gift from Prof. Yoshinori Ohsumi) and then probed with secondary anti-mouse-IgG (Bio-Rad, 1:10,000) and anti-rabbit-IgG (Bio-Rad, 1:10,000) antibodies conjugated to HRP and were developed on a gel documentation system (G: Box chemi XT4, Syngene).

### Statistics

All statistical analysis was performed using GraphPad Prism. To calculate significance levels two-way ANOVA and Student's *t*-tests were used. The mean±s.e.m. is shown in all graphs.

## Supplementary Material

Supplementary information
