# Supplementary material for: Septins are involved at the early stages of macroautophagy in S. cerevisiae
Source: J Cell Sci. 2018 Feb 15;131(4):jcs209098. doi: 10.1242/jcs.209098 (PMC5868950; doi:10.1242/jcs.209098)
Supplement: Supplementary information [file joces-131-209098-s1.pdf]

Supplementary information

Septins are involved at the early stages of macroautophagy in *S. cerevisiae*

Gaurav Barve[1], Shreyas Sridhar[1], Amol Aher[1], Mayurbhai H. Sahani[1], Sarika Chinchwadkar[1], Sunaina Singh[1], Lakshmeesha K.N.[1], Michael A. McMurray[2] and Ravi Manjithaya*

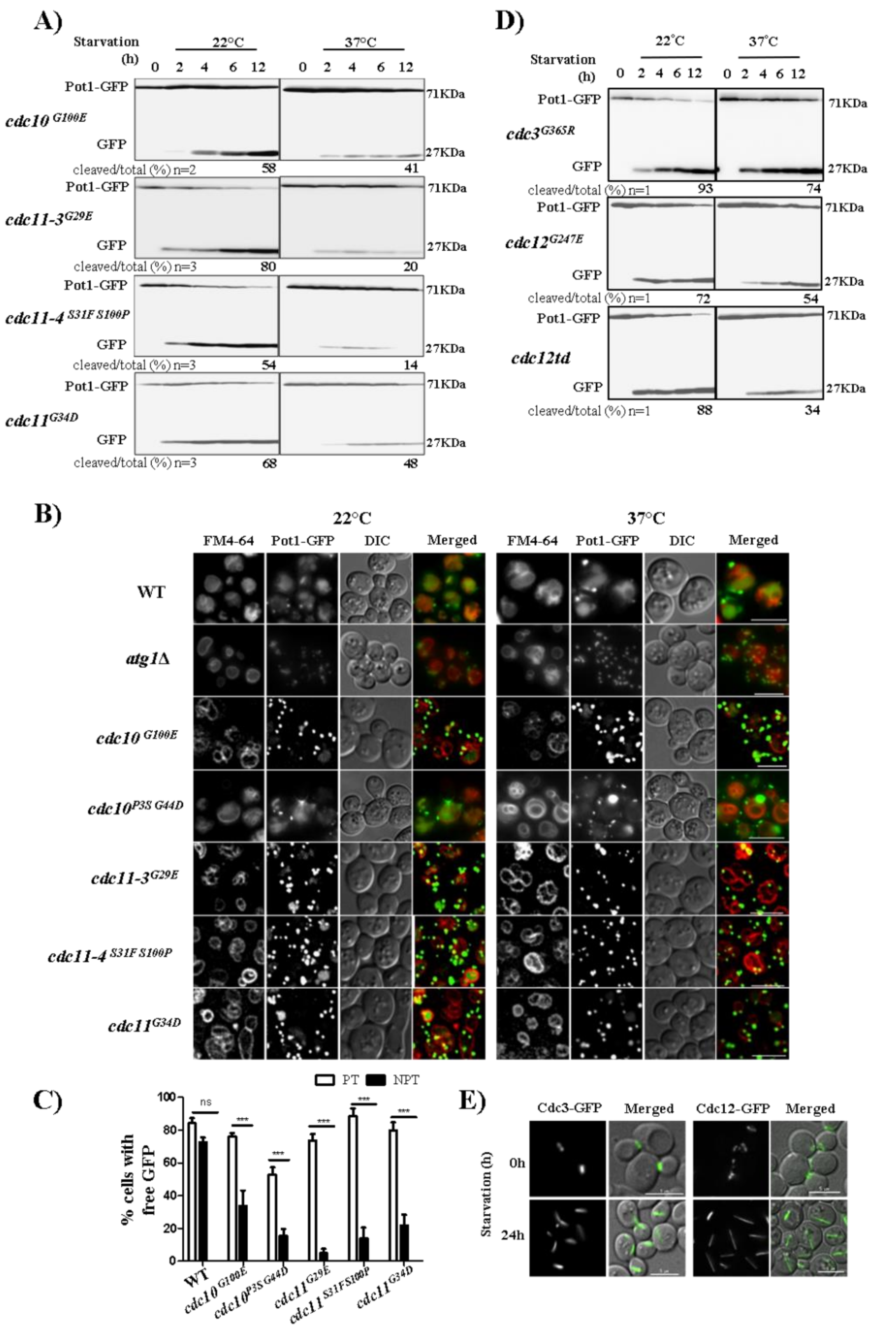

Figure S1. Pexophagy was affected in septin Ts^{-} mutants.

(A) Pexophagy assay in septin Ts^{-} mutants. WT and Ts^{-} mutant cultures (0.6 to 0.8 OD) expressing Pot1-GFP were grown in oleate medium and transferred to starvation medium (3 OD/ml) and incubated at permissive (22°C) and non-permissive temperatures (37°C). Cells were collected at indicated time points and analyzed by Western blotting. **(B)** Pexophagy assay scored

Journal of Cell Science • Supplementary information

using microscopy. Cells were grown as mentioned in Fig. S1A 6 h at 22°C and 37°C and images were taken. All images are single Z-stacks. **(C)** The number of cells showing free GFP inside the vacuole labelled with FM4-64 was quantitated. A total of 100 cells were quantitated (***$p<0.001$, 22°C versus 37°C, Two-way ANOVA). **(D)** Pexophagy in *cdc3*^{G365R}, *cdc12*^{G247E}, *cdc12td*. Assay was performed as mentioned in **Fig. S1A.** The number below each blot is percentage of cleaved GFP from Pot1-GFP fuse protein where "n" indicates number of experiments. For quantification, ratio of GFP/(Pot1-GFP+GFP) was calculated for indicated time point (12h) and was then converted into percentage. Asterisks indicate non-specific bands. **(E)** Localization of Cdc3-GFP and Cdc12-GFP in nutrient rich (0 h) and starvation condition (24 h). Cells were grown as mentioned in **Fig. 1B.** Scale bars: 5 µm.

Journal of Cell Science • Supplementary information

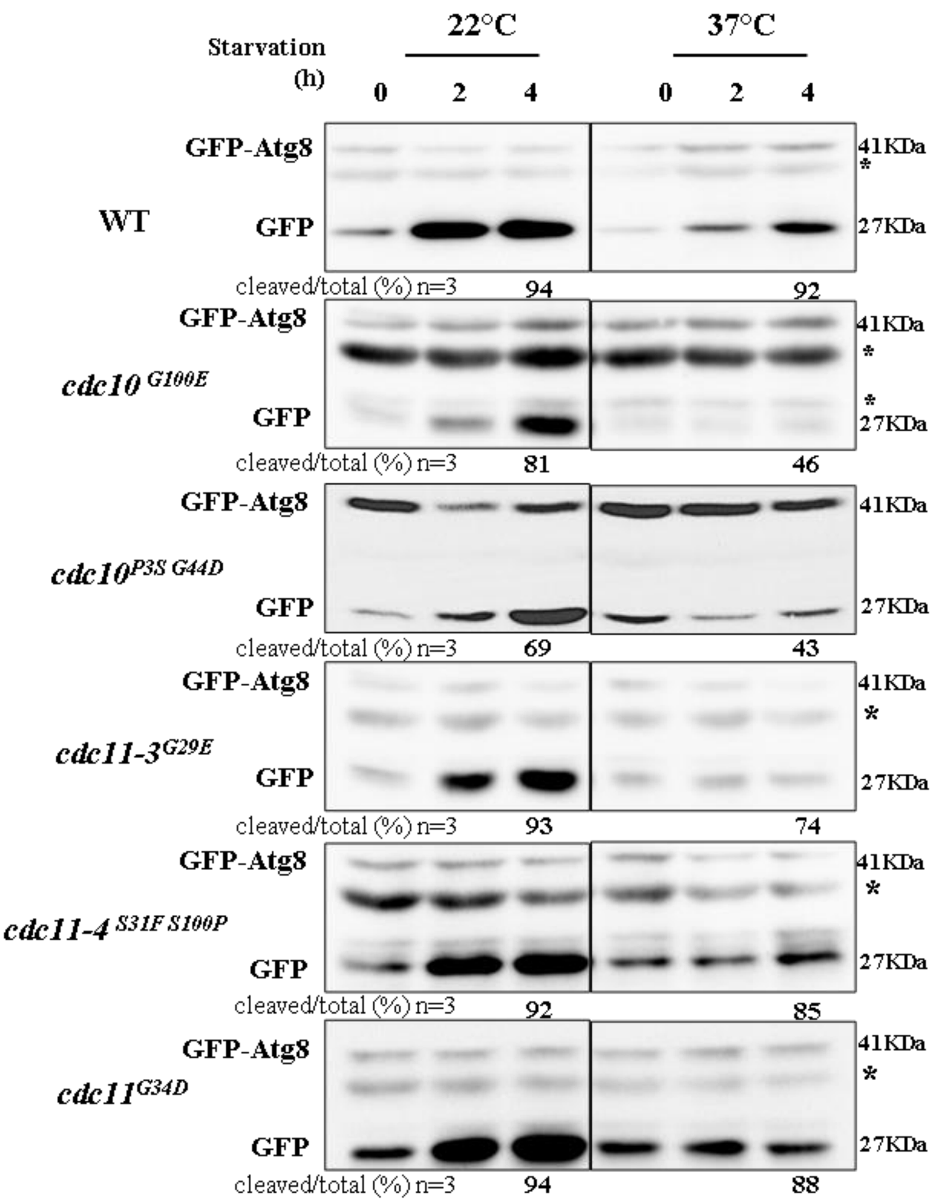

Figure S2. General autophagy was affected in septin Ts$^-$ mutants.

(A) General autophagy assay in Ts$^-$ mutants. WT and Ts$^-$ mutant cultures (0.6 to 0.8 OD) expressing GFP-Atg8 were grown in SD-Ura medium and transferred to starvation medium (3 OD/ml). Cells were incubated at 22°C and 37°C and were collected at indicated time points and analysed by Western blotting. The number below each blot is percentage of cleaved GFP from GFP-Atg8 fuse protein where “n” indicates number of experiments. For quantification, ratio of GFP/(GFP-Atg8+GFP) was calculated for indicated time point (4 h) and was then converted into percentage. Asterisks indicate non-specific bands.

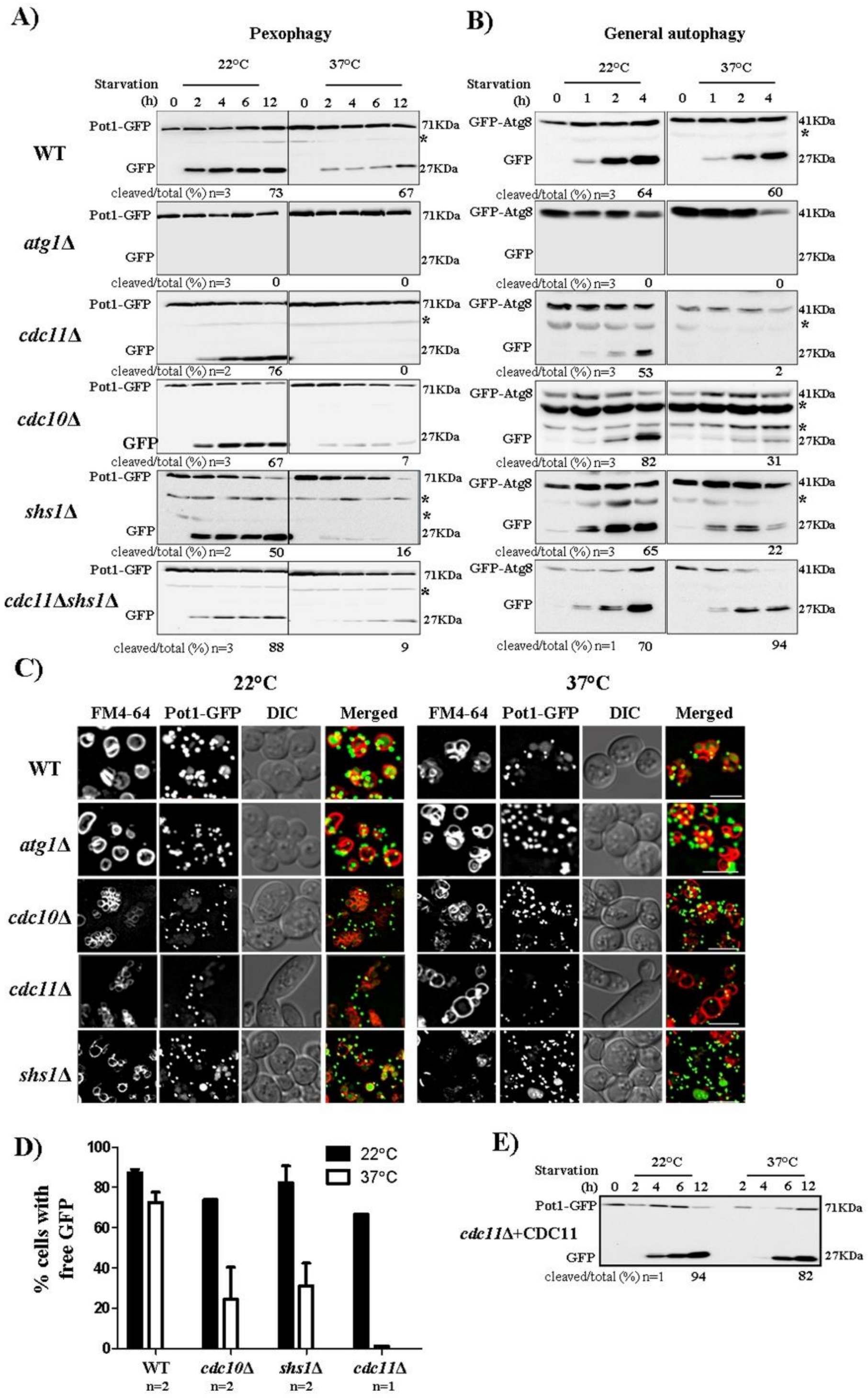

Figure S3. Pexophagy and general autophagy were affected in septin knockout mutants.

(A) Pexophagy assay in septin deletion mutants. WT, *atg1*Δ and septin deletion mutants expressing Pot1-GFP were grown as for **Fig. S1A**. **(B)** General autophagy assays in deletion

Journal of Cell Science • Supplementary information

mutants. WT and septin deletion mutants were processed as for **Fig**. **S2**. **(C)** Pexophagy assay scored using microscopy. For microscopy, cells were incubated as mentioned in **Fig. S1B**. Asterisks indicate the non-specific bands. **(D)** A total of 50 cells showing free GFP inside the vacuole labeled with FM4-64 was quantitated and the mean of the two individual experiments is plotted (except for *cdc11*Δ). **(E)** Pexophagy assay was rescued when WT CDC11 copy was expressed in *cdc11*Δ cells. Experiment was performed as mentioned in **Fig. S1A.** Scale bar 5 µm.

Journal of Cell Science • Supplementary information

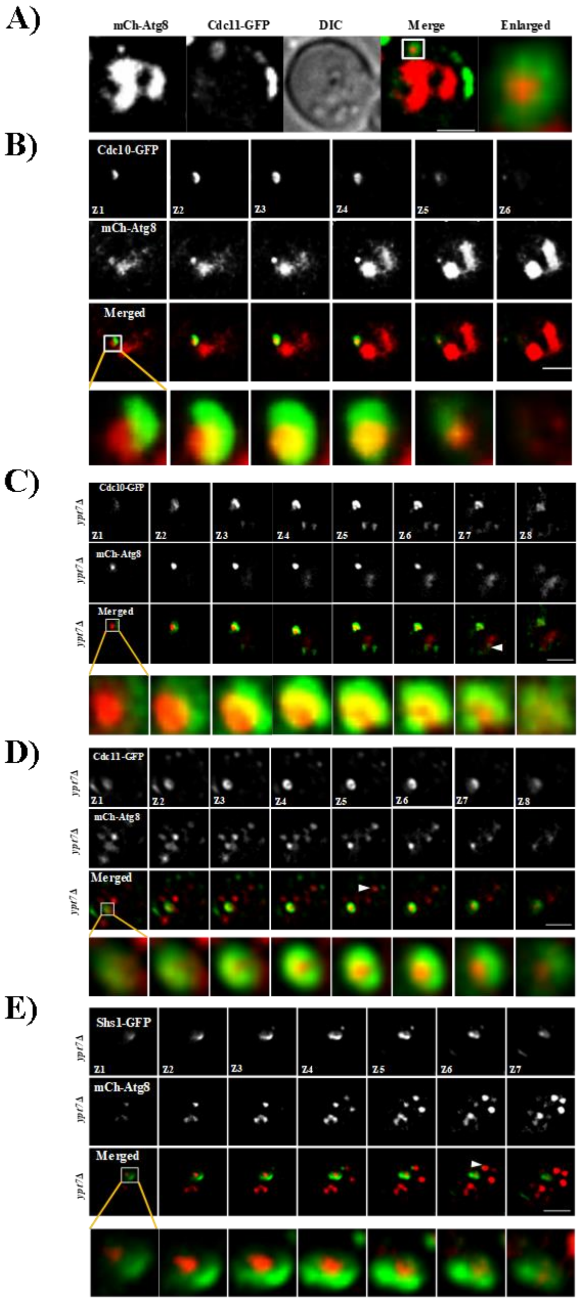

Figure S4. Formation of non-canonical ring around autophagosomes.

Formation of non-canonical ring around mCh-Atg8 by **(A)** Cdc11-GFP. **(B)** Cdc10-GFP. WT cells expressing either Cdc10-GFP or Cdc11-GFP were grown till 0.6-0.8 OD, transferred to starvation media and were imaged after 4 h. Z-sections of 0.2µm each are shown. **(C), (D) and (E)** Cdc10-GFP, Cdc11-GFP and Shs1-GFP respectively showed formation of non-canonical ring around mCh-Atg8 in *ypt7*Δ cells. Apart from autophagosomes that co-localize with the non-canonical rings, all the three septins also showed co-localization with autophagosomes at other sites (shown by white arrow heads). Scale bar 2 µm.

Journal of Cell Science • Supplementary information

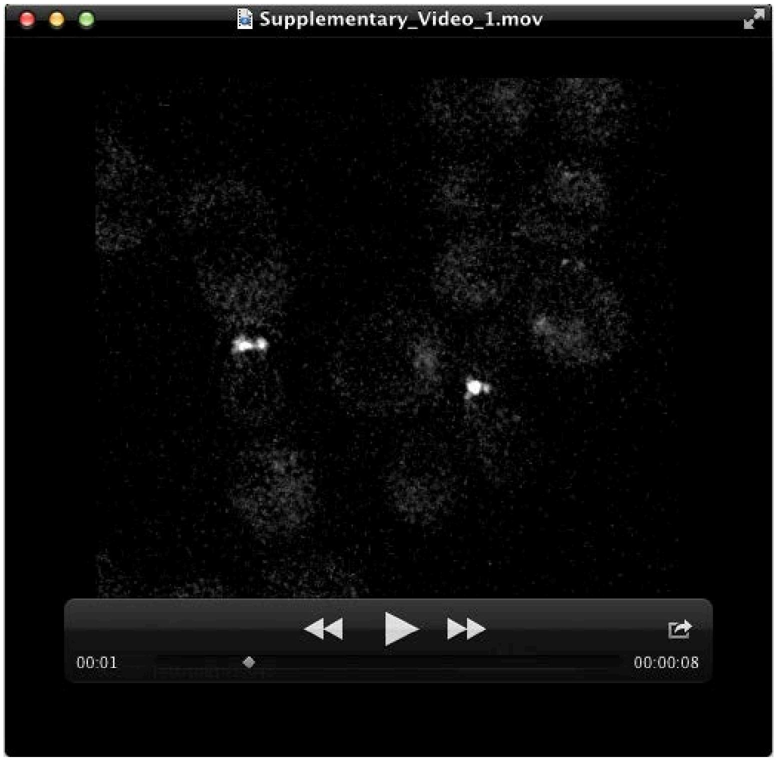

Supplementary Video 1. Dissociation of *cdc10(G100E)-GFP* from the bud-neck into cytoplasm during starvation. *cdc10(G100E)-GFP* cells expressing mCh-Atg8 were grown in SD-Ura medium at 22°C and the logarithmically growing cells were then transferred into starvation media and incubated at 22°C and 37°C. Immediately after the transfer into starvation media, 1 OD cells were collected as 0 h time point and were mounted on agarose pads (made in SD-N medium). Time-lapse was carried out for 30 min with an interval of 2 min at 22°C. Only GFP channel is shown. Scale bar 5 µm.

Journal of Cell Science • Supplementary information

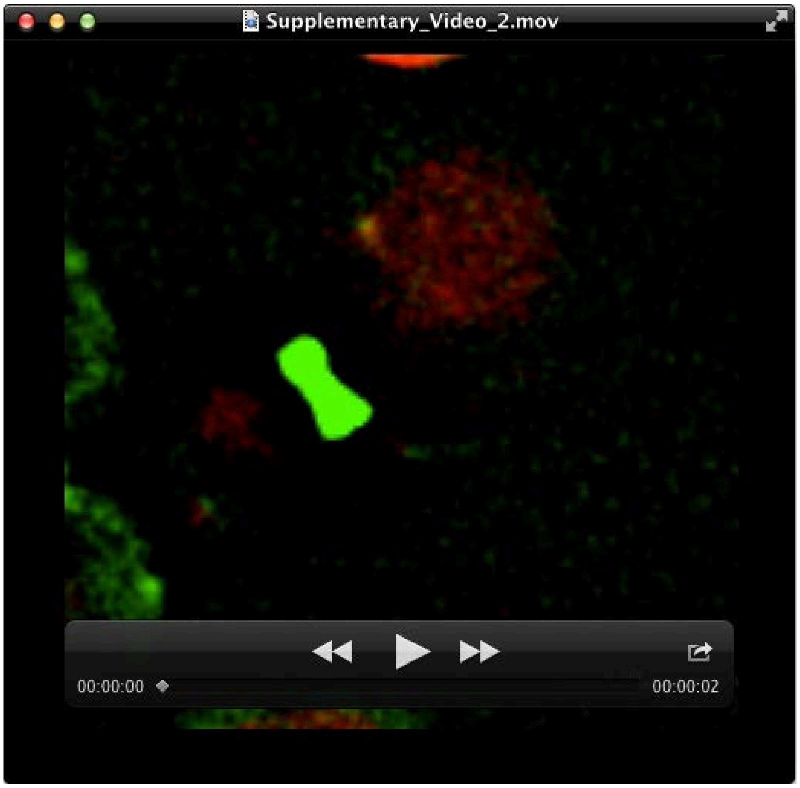

Supplementary Video 2. Cdc10-GFP is shown as representative of the three septins. Dynamic interaction of Cdc10-GFP with mCh-Atg8 is shown here. Cells were grown in SD-Ura medium at 30°C and the logarithmically growing cells were then transferred into starvation media. After 4 h incubation in starvation media, 1 OD cells were collected and were mounted on agarose pads (made in SD-N medium). Time-lapse was carried out for 20 min with an interval of 4 min at RT Scale bar 2 μm.

Journal of Cell Science • Supplementary information

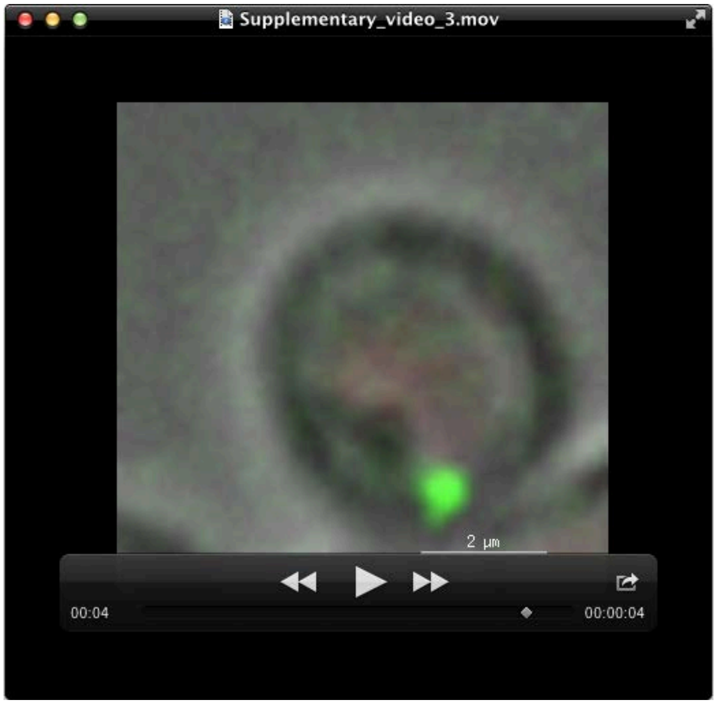

Supplementary Video 3. BiFC study that shows Cdc10 transiently interact with Atg9 at PAS (Ape1-RFP). Cells expressing Cdc10-Vc, Atg9-Vn and Ape1-RFP were grown in SD-his medium at 30°C and the logarithmically growing cells were then transferred into starvation media. After 5 h incubation in starvation media, 1 OD cells were collected and were mounted on agarose pads (made in SD-N medium). Time-lapse was carried out for 100 min with an interval of 10 min at RT. Scale bar 2 µm.

Supplementary Table S1

Click here to Download Table S1

Supplementary Table S2

Click here to Download Table S2

Journal of Cell Science • Supplementary information
